# Cost effectiveness of personalized treatment in women with early breast cancer: the application of OncotypeDX and Adjuvant! Online to guide adjuvant chemotherapy in Austria

**DOI:** 10.1186/s40064-015-1440-6

**Published:** 2015-12-01

**Authors:** B. Jahn, U. Rochau, C. Kurzthaler, M. Hubalek, R. Miksad, G. Sroczynski, M. Paulden, M. Kluibenschädl, M. Krahn, U. Siebert

**Affiliations:** Institute of Public Health, Medical Decision Making and Health Technology Assessment, Department of Public Health, Health Services Research and Health Technology Assessment, UMIT - University for Health Sciences, Medical Informatics and Technology, Eduard Wallnoefer Center 1, A-6060 Hall i.T, Austria; Division of Public Health Decision Modelling, Health Technology Assessment and Health Economics, ONCOTYROL - Center for Personalized Cancer Medicine, Innsbruck, Austria; Department of Obstetrics and Gynaecology, Medical University Innsbruck, Innsbruck, Austria; Harvard Medical School, Beth Israel Deaconess Medical Center, Boston, MA USA; Toronto Health Economics and Technology Assessment (THETA) Collaborative, University of Toronto, Toronto, ON Canada; Department of Emergency Medicine, University of Alberta, Edmonton, AB Canada; Center for Health Decision Science, Department of Health Policy and Management, T.H.Chan Harvard School of Public Health, Boston, MA USA; Institute for Technology Assessment and Department of Radiology, Harvard Medical School, Massachusetts General Hospital, Boston, MA USA

**Keywords:** 21-Gene assay, Genetic testing, Adjuvant! Online, Cost effectiveness, Decision-analytic model, Discrete event simulation

## Abstract

A Breast Cancer Outcomes model was developed at the ONCOTYROL research center to evaluate personalized test-treatment strategies in Austria. The goal was to evaluate the cost-effectiveness of a new 21-gene assay (ODX) when used in conjunction with the Adjuvant! Online (AO) decision aid to support personalized decisions about use of adjuvant chemotherapy in early-stage breast cancer patients in Austria. We applied a validated discrete-event-simulation model to a hypothetical cohort of 50 years old women over a lifetime horizon. The test-treatment strategies of interest were defined using three-letter acronyms. The first (second, third) letter indicates whether patients with a low (intermediate, high) risk according to AO were tested using ODX (*Y* yes, *N* no). The main outcomes were life-years gained, quality-adjusted life-years (QALYs), costs and cost effectiveness. Robustness of the results was tested in sensitivity analyses. Results were compared to a Canadian analysis conducted by the Toronto Health Economics and Technology Assessment Collaborative (THETA). Five of eight strategies were dominated (i.e., more costly and less effective: NNY, NYN, YNN, YNY, YYN). The base-case analysis shows that YYY (ODX provided to all patients) is the most effective strategy and is cost effective with an incremental cost-effectiveness ratio of 15,700 EUR per QALY gained. These results are sensitive to changes in the probabilities of distant recurrence, age and costs of chemotherapy. The results of the base-case analysis were comparable to the THETA results. Based on our analyses, using ODX in addition to AO is effective and cost effective in all women in Austria. The development of future genetic tests may require alternative or additional test-treatment strategies to be evaluated.

**Electronic supplementary material:**

The online version of this article (doi:10.1186/s40064-015-1440-6) contains supplementary material, which is available to authorized users.

## Background

Breast cancer accounts for 28 % of all malignancies in Austrian women (Statistik Austria 2012). Based on current projections, seven of every 100 Austrian girls born in 2009 will develop breast cancer by the age of 75 (Statistik Austria 2012). Aside from a small percentage of familial breast cancer syndromes, risk factors include age, early menarche, late menopause, and obesity [Arbeitsgemeinschaft für gynäkologische Onkologie (AGO) der Österreichischen Gesellschaft für Gynäkologie und Geburtshilfe (OEGGG) 2012]. Currently, various treatment strategies are available [Leitlinienprogramm Onkologie (OL) 2012]. When possible, surgical resection offers a potential cure. Adjuvant treatment after surgery with radiation and/or systemic therapy (including chemotherapy and/or hormonal therapy) depends on the surgical approach, stage of the disease, hormone receptor status [e.g., estrogen receptor (ER) and progesterone receptor (PR) status], postmenopausal status, HER2/neu status, pathologic findings and co-morbidities. In part due to the length of treatment and potential severe and lethal side effects, the use of adjuvant chemotherapy has been heavily investigated in terms of its impact on risk of recurrence and overall survival.

In particular for women with lymph node negative, ER positive early-stage breast cancer, the decision about whether to treat with adjuvant chemotherapy is complex and uncertain. Adjuvant chemotherapy can be beneficial for women at higher risk of a distant recurrence, but can cause more harm for low risk patients. There are several prognostic tests available that can identify women who are most likely to benefit from adjuvant therapy. For example, Adjuvant! Online (AO) is a free, web-based decision aid designed to help patients and clinicians understand individual risk, the impact of systemic therapy. AO estimates breast cancer-specific mortality and recurrence after surgery for various chemotherapy and hormonal treatment options based on the patient’s age, comorbidities, estrogen receptor status, tumor size, tumor grade and number of positive lymph nodes (Adjuvant! Online Inc 2012). MammaPrint^®^ and OncotypeDX^®^ are gene expression assays that quantify the risk of distant disease recurrence (Agency for Healthcare Research and Quality 2012).

We evaluated the benefit-harm balance and cost effectiveness of the 21-gene assay OncotypeDX^®^ [Genomic Health Inc., Redwood City, CA, USA (ODX)] because it was considered potentially useful for adjuvant chemotherapy decision-making by 84 % of the experts at the 2011 St. Gallen Consensus Conference (Gnant et al. 2011). In 2013, the European Society for Oncology (ESMO) concurred that in cases of uncertainty regarding adjuvant chemotherapy (after consideration of other tests), gene expression, assays such as MammaPrint^®^ or OncotypeDX^®^ may be used. The analytical and clinical test validity of this assay convinced the majority of the IMPACT 2012 Working Group members in its clinical use (Azim et al. 2013). However, the 21-gene assay is not currently offered in Austria.

Several studies have evaluated the impact of ODX on treatment decisions as compared to common clinical and pathological criteria such as the National Comprehensive Cancer Network (NCCN) guidelines, the NSABP B-20 study or Adjuvant! Online (AO) (Adjuvant! Online Inc 2012). Most studies have identified an impact on decisions and changes in use of chemotherapy in up to 50 % of all patients (Asad et al. 2008; Henry et al. 2009; Klang et al. 2010; Lo et al. 2010; Oratz et al. 2007, 2011). For the most part, recommendations have changed from chemotherapy plus hormonal therapy to hormonal therapy alone in patients with ER-positive and LN-negative breast cancer (Henry et al. 2009; Lo et al. 2010; Liang et al. 2007). In these patients, chemotherapy and its associated adverse events may be avoided. In addition, increased confidence among medical oncologists and decreased patient anxiety were reported with the use of ODX (Lo et al. 2010) .

The cost effectiveness of using ODX has been evaluated in several studies (Klang et al. 2010; Hall et al. 2012; Kondo et al. 2008, 2011; Lyman et al. 2007; Paulden et al. 2013; Reed et al. 2009; Tsoi et al. 2010; Ward et al. 2013; Yang et al. 2012; Hornberger et al. 2005). However, physicians may want to consider clinicopathological factors (like in AO) along with genomic test results, especially when the risk classification with AO is validated and free of charge. We found only two studies that considered the combination of both tests (Paulden et al. 2013; Reed et al. 2009). These studies did not cover Austrian treatment patterns or costs.

Therefore, the aim of our study was to evaluate the effectiveness and the cost effectiveness of ODX when it is used or not used in conjunction with the AO score to guide decisions about use of adjuvant chemotherapy women diagnosed with ER and/or PR positive, HER-2/neu negative, and lymph node negative breast cancer in Austria.

## Methods

### Model design and assumptions

We applied a previously published and validated decision-analytic model to estimate life years (LY) and quality-adjusted life years (QALY) (Jahn et al. 2012a, b) (Jahn et al. 2015). In addition, we also estimated the number of adverse drug events due to chemotherapy, number of patients with a distant recurrence, number of patients who die from a distant recurrence, long term costs as well as incremental cost-effectiveness ratios (ICER) for each of the eight strategies being evaluated (see next section). In the base-case analysis, a hypothetical cohort of 50-year old women diagnosed with ER and/or PR positive, HER-2/neu negative, and lymph node negative breast cancer was evaluated over their remaining lifetime. The societal perspective in the Austrian health care context was adopted. Only direct costs were considered to avoid double counting (Gold et al. 1996). For our Oncotyrol model, we applied a discrete event simulation model (DES) approach using ARENA (version 13.90.00000, Rockwell Automation). Our choice was guided by the ISPOR-SMDM guidelines that take into account that individual patient pathways are determined by multiple characteristics and test results, while considering time-dependent functional relationships, and recording of individual patient pathways (Karnon et al. 2012; Caro et al. 2012).

### Model structures

**Fig. 1:**
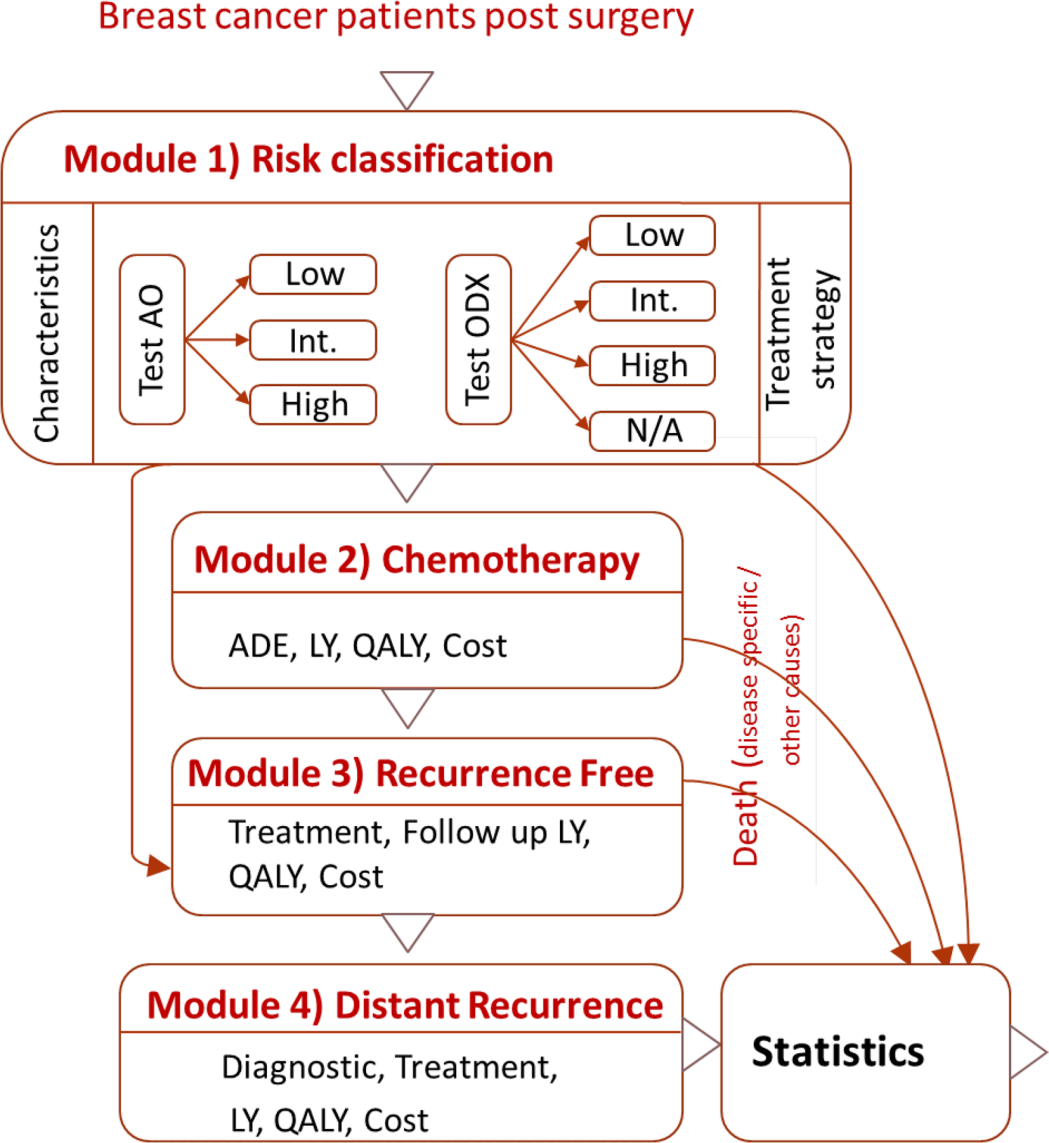
Schematic model structure (ER/PR + : estrogen and/or progesterone receptor positive, LY: life years, QALY: quality adjusted life years, ADE: adverse drug event, Int.: intermediate, AO: Adjuvant!Online, ODX: *Oncotype*DX, N/A: not applied) Source: Jahn et al. (2015). Lessons Learned from a Cross-Model Validation between a Discrete Event Simulation Model and a Cohort State-Transition Model for Personalized Breast Cancer Treatment. Medical Decision Making (published ahead of print). Copyright © 2015 by Society for Medical Decision Making. Reprinted by permission of SAGE Publications, Inc.

In this individual-level computer simulation, hypothetical patients were projected over a lifelong analytic time horizon after they had received surgery. The model included 4 parts: first, patients were assigned their characteristics and test results (Module 1—risk classification); second, chemotherapy was provided if necessary (Module 2—chemotherapy); third, the time patients remained free from a distant recurrence was considered (Module 3—recurrence free); and fourth, distant recurrence was considered (Module 4—recurrence). Finally, a statistical module provided a summary of the model outcomes (Fig. 1).

In Module 1, we evaluated eight two-test strategies to determine if chemotherapy should be given. We assumed that each patient was assigned an individualized breast cancer specific mortality (BCSM) risk based on the AO assessment. (Adjuvant! Online Inc 2012): ‘low’ (BCSM < 9 %), ‘intermediate’ (9 % ≤ BCSM < 17 %) or ‘high’ (BCSM ≥ 17 %) risk (Bryant 2005). The BCSM risk assigned by AO was then used to determine if genomic testing with ODX would be pursued. Because genomic testing results in additional costs and because it is recommended that ODX may be used in conjunction with all clinical and pathologic factors in cases where the decision about use of chemotherapy is difficult (Azim et al. 2013; al 2013), we assume a sequential application (AO first, followed by ODX). ODX provides a recurrence risk score between 0 and 100, in which ‘low’ is considered RS < 18, ‘intermediate’ 18 ≤ RS < 30 and ‘high’ (RS ≥ 30) (Paik et al. 2004). The eight test-treatment strategies evaluated are described using three letters: NNN, YYY, NNY, NYN, YNN, YYN, YNY and NYY. The first letter indicates whether patients with a low risk according to AO were tested using the 21-gene assay (Y-yes; N–no), the second and the third letters provide this information for AO intermediate and high risk patients, respectively (e.g., NYN means that only patients with an AO intermediate risk are tested with the 21-gene assay). Adjuvant chemotherapy was then provided according to the results of both tests (see Table 1). Patients in the model move to Module 2 (adjuvant chemotherapy) or directly to Module 3 (follow-up) as appropriate. In Module 2, chemotherapy and associated adverse drug events (neutropenia, fever, infections, pain, nausea, gastrointestinal complications) were modeled. After the administration of chemotherapy, patients were considered recurrence-free and move to Module 3. In Module 3, each patient was followed until death from other causes or distant recurrence. Within the first 3 years, patients are assumed to have quarterly visits, thereafter biannually and after 5 years, annually. The regular visits consist of a clinical examination and mammography. Within the first 5 years, all patients receive aromatase inhibitors. In the case of a distant recurrence, the associated diagnostic work-up and treatment are modeled in Module 4. At any point in time, patients may die from other causes. In addition, patients receiving chemotherapy may die from adverse events and patients with metastatic disease may die from breast cancer. Within the simulation, information on patients’ remaining lifetime and quality-adjusted lifetime, adverse drug events, and costs are accumulated and final outcomes are computed at the end of their simulated lifetime.

**Table 1:** Model parameter overview

Parameters	Values	Sources
Proportion of patients assigned to each risk group		
Adjuvant! Online low risk	52.99 %	Paulden et al. (2013)
21-Gene assay low risk	32.34 %	
21-Gene assay int. risk	12.57 %	
21-Gene assay high risk	8.08 %	
Adjuvant! Online int. risk	18.71 %	
21-Gene assay low risk	8.53 %	
21-Gene assay int. risk	3.59 %	
21-Gene assay high risk	6.59 %	
Adjuvant! Online high risk	28.29 %	
21-Gene assay low risk	9.73 %	
21-Gene assay int. risk	6.14 %	
21-Gene assay high risk	12.43 %	
Proportion of patients in each risk group provided adjuvant chemotherapy^a^		
Adjuvant! Online low risk	0 %	Lo et al. (2010); Medical University Innsbruck (2012)
21-Gene assay low risk	0 %	
21-Gene assay int. risk	17.62 %	
21-Gene assay high risk	63.44 %	
Adjuvant! Online int. risk	55.06 %	
21-Gene assay low risk^a^	13.73 (T1, base case)/(0 % T2, SA)	
21-Gene assay int. risk	36.56 %	
21-Gene assay high risk	98.61 %	
Adjuvant! Online high risk	57.57 %	
21-Gene assay low risk	13.72 %	
21-Gene assay int. risk	36.65 %	
21-Gene assay high risk	99.73 %	
*Risk of hospital visit due to toxicity*	17.04 %	Tilak Financial Department and Cost Data Report (2012)
Cause of hospital visits due to toxicity		
Neutropenia/fever/infections	53.56 %	Medical University Innsbruck (2012)
Pain & pain management	7.51 %	
Nausea/vomiting/dehydration	6.02 %	
Gastrointestinal tract	5.64 %	
10 year risk for distant recurrence without chemotherapy		
Adjuvant! Online low risk	5.39 %	Paulden et al. (2013)
21-Gene assay low risk	2.61 %	
21-Gene assay int. risk	3.84 %	
21-Gene assay high risk	18.91 %	
Adjuvant! Online int. risk	20.36 %	
21-Gene assay low risk	4.24 %	
21-Gene assay int. risk	14.90 %	
21-Gene assay high risk	44.23 %	
Adjuvant! Online high risk	24.12 %	
21-Gene assay low risk	4.24 %	
21-Gene assay int. risk	14.90 %	
21-Gene assay high risk	44.23 %	
10 year risk for distant recurrence with chemotherapy		
Adjuvant! Online low risk	5.68 %	Paulden et al. (2013)
21-Gene assay low risk	4.98 %	
21-Gene assay int. risk	5.62 %	
21-Gene assay high risk	8.58 %	
Adjuvant! Online int. risk	7.35 %	
21-Gene assay low risk	5.79 %	
21-Gene assay int. risk	8.18 %	
21-Gene assay high risk	8.91 %	
Adjuvant! Online high risk	7.68 %	
21-Gene assay low risk	5.79 %	
21-Gene assay int. risk	8.18 %	
21-Gene assay high risk	8.91 %	
*Risk of mortality due to toxicity from chemotherapy*	0.1 %	Medical University Innsbruck (2012)
*Median life expectancy following distant recurrence* (*months*)	25.8	Medical University Innsbruck (2012)
*Risk of mortality due to other causes*	Life table	Statistik Austria (2012)
Costs (inflated to 2011 Euros)		
21-Gene assay	3180	Jahn, Personnel email-communication with manufacturer (2012, unpublished)
Costs for chemotherapy		
Echocardiography (one time)	28	Tilak Financial Department and Cost Data Report (2012)
Chest radiography (one time)	23	
Port implantation (one time)	550	
Laboratory test (per cycle of chemotherapy)	46.5	
Blood panel (per week for 6 months)	3.75	
Human resources (per cycle of chemotherapy)	48	
Hospitalization (3 days)	620	
*Total additional costs for* *chemotherapy* (6 months)	2,089.5	
*Total costs for FEC* (Fluorouracil 500 mg/m^2^, Cyclophospamid 600 mg/m^2^, Epirubicin 90 mg/m^2^)	672.16	
*Total costs for DOC* (Docetaxel 75 mg/m^2^)	1042.5	
Pegfilgrastim 6 mg	1175.57	
Tropisetron-Hydrochlorid 5 mg 5 pills	85.90	
*Total costs for chemotherapy* (*Additional costs, FEC, DOC, Pegfilgrastim, Tropisetron-Hydrochlorid, 6* *months*)	11,372.96	*Derived from* Tilak Financial Department and Cost Data Report (2012); Medical University Innsbruck (2012)
Follow up costs for the first 5 years after chemo therapy (costs per month/treatment)		
Anastozol 1 mg	73.7	Tilak Financial Department and Cost Data Report (2012)
Lertozolum 2.5 mg	*101.2*	
Exemestanum 25 mg	*75.87*	
*Other treatment costs for the first 5* *years after chemo*	*5016.8*	*Derived from* Tilak Financial Department and Cost Data Report (2012); Medical University Innsbruck (2012)
Mammography	*32*	Tilak Financial Department and Cost Data Report (2012)
Examination	*85.5*	
*Follow up costs for the first 5* *years after chemo per month*	*21.54*	*Derived from* Tilak Financial Department and Cost Data Report (2012); Medical University Innsbruck (2012)
Follow up Costs after the first 5 years after chemo therapy (costs per month/treatment)		
*Follow up costs per month*	*9.79*	*Derived from* Tilak Financial Department and Cost Data Report (2012); Medical University Innsbruck (2012)
Costs of diagnosing distant recurrence		
Total costs of diagnosis of distant recurrence	*248.5*	*Derived from* Tilak Financial Department and Cost Data Report (2012); Medical University Innsbruck (2012)
Costs of treating distant recurrence		
Total costs per 25.8 months	32,015.26	Tilak Financial Department and Cost Data Report (2012)
Treatment of non-fatal chemotherapy toxicity		
Neutropenia/fever/infections	5231.46	Ontario Case Costing Initiative (2011); Medical University Innsbruck (2012)
Pain management	3270.66	
Nausea/vomiting/dehydration	3173.45	
Gastrointestinal tract	5169.31	
Treatment of fatal toxicity	36,260	Walter (2012)
Utility weights		
First year following diagnosis (while on hormone therapy)	0.744	Lidgren et al. (2007)
First year following diagnosis (while on chemotherapy)	0.620	
Second and following years prior to distant recurrence	0.779	
Following distant recurrence	0.685	
Dead	0	

^a^Parameter values differ for the two treatment strategies T1 (base case)/T2 (sensitivity analysis)

The model structure and a comprehensive model validation process are described in more detail elsewhere (Jahn et al. 2012a, b, 2015).

### Model parameters

#### Clinical parameters

Table 1 provides an overview of model parameters and sources.

The proportion of patients assigned to each of the 12 risk groups according to AO and ODX was based on a retrospective study (Paulden et al. 2013). The provision of chemotherapy within the twelve risk groups was based on a prospective study in a North American population (Lo et al. 2010) and adapted based on Austrian expert opinion (Medical University Innsbruck 2012) for the AO low risk groups. Time to recurrence for different risk classes was derived from Paulden et al. (2013). Because there are no published prospective studies that report distant recurrences conditional on 21-gene assay or AO risk, Paulden et al. (2013) used findings from a retrospective analysis based on a subset of the NSABP B-14 study (Bryant 2005) and from a subset of the NSABP B-20 study (Paik et al. 2006).

Fatal toxicity of chemotherapy includes 0.1 % of patients that will subsequently develop acute myeloid leukemia.

For patients with distant recurrence, we assumed that the probability of death due to breast cancer was identical in all patients regardless of the ER/PR status or patients′ personal cancer history. Median survival for these patients was estimated as 25.8 months (Medical University Innsbruck 2012). We assumed that the time to recurrence in recurrence-free patients and the time to death in patients with recurrence was exponentially distributed.

All-cause mortality was extrapolated using life tables from Statistics Austria (2012).

### Cost data

The price of the 21-gene assay was adapted from the manufacturer’s suggested retail price for Austria (Jahn, Personnel email-communication with manufacturer 2012, unpublished). Direct costs for chemotherapy, cancer follow-up, and diagnosis and treatment of recurrent malignancies were based on internal calculations of the financial department of the Tyrolienne Hospital Operating Company (TILAK) (Tilak Financial Department and Cost Data Report 2012) and expert opinion from the Innsbruck Medical University (Medical University Innsbruck 2012). Drug costs were based on pharmacy hospital prices.

Costs for chemotherapy included the costs of chemotherapeutic agents, other supportive medications such as pegfilgrastim and tropisteron, hospitalization, laboratory studies, and human resources. It was assumed that all patients receive the current standard of care in terms of chemotherapeutic agents. At present, this is three cycles of FEC (5-Fluorouracil, Epirubicin, Cyclophosphamid) followed by 3 cycles of DOC (Docetaxel) (Hubalek 2010).

All patients also received an aromatase inhibitor (anastozol or lertozolum or aromasin) for 5 years. This was begun immediately in all cases except in those who received chemotherapy, in which case the aromatase inhibitor was started following the completion of adjuvant chemotherapy.

Underlying assumptions regarding the treatment of distant recurrence were derived from chart reviews by a senior gynecologist at Innsbruck Medical Hospital.

Costs associated with adverse drug events of chemotherapy were based on published data (Ontario Case Costing Initiative 2011) and adapted according to Austrian experts (Medical University Innsbruck 2012). Treatment of fatal toxicity included treatment of acute myeloid leukemia. Cost data were evaluated based on German DGHO treatment guidelines (DGHO guidelines 2013). Cost were derived from or inflated to Euros 2011. Costs and health outcomes were discounted at 5 % (Walter and Zehetmayr 2006).

### Utility data

Utility weights for the different breast cancer states were derived from a recent cross-sectional observational study (Lidgren et al. 2007) using the EQ-5D, a standardized, non-disease-specific instrument for describing and valuing health states (while on hormone therapy, while on chemotherapy, prior to distant recurrence, following distant recurrence).

## Analysis

### Base-case analysis

We estimated discounted LYs, QALYs, number of patients who experienced adverse drug events, disease recurrence, costs, and ICERs (additional costs per QALY gained). For the cost-effectiveness results, we excluded dominated strategies. These are strategies that provide fewer QALYs at a higher cost. We also excluded strategies due to extended dominance. Strategies are extendedly dominated if there is a more expensive strategy with a lower ICER. (Cantor 1994).

The cost-effectiveness results are displayed on a cost-effectiveness plane. The resulting line that connects the most efficient strategies is called the efficiency frontier. Strategies that lie on the efficiency frontier are relevant for decision makers, whereas strategies below the line are considered dominated and inefficient.

Finally, incremental cost-effectiveness ratios of strategies on the efficiency frontier are compared to the relevant threshold. In Austria, there is currently no explicit cost-effectiveness threshold for the adoption of health technologies. Thresholds applied in other countries vary and are rarely cancer specific (Schwarzer et al. 2015). For example, in Canada the chair of the Ontario expert review committee suggested an oncology-specific ceiling threshold value of C$75,000 (this is equivalent to $US65,914 for 2009). NICE provided a general threshold in 2012 of £18,317/QALY (US$25,435) that can be altered based on other factors.

### Sensitivity analyses

We performed several one-way sensitivity analyses to account for parameter uncertainty (“second-order uncertainty”). This relates to the fact that the probabilities applied in the model are themselves uncertain: for example, values from clinical studies have confidence intervals. (Briggs et al. 2012a, b). The results are displayed in a modified tornado diagram that shows the variation in outcomes according to one parameter on a horizontal bar. Along the vertical axis, all relevant parameters are shown. The outcome of the base-case is indicated by a vertical line cutting through all horizontal bars. The longest bar reflects the most uncertain parameter (Briggs et al. 2012, b). We applied a content specific order of the parameters (bars), that is: age (40; 50; 70), discount rate (0; 2.5 %; 5 %), cost of chemotherapy (±10 %), cost of ODX test set (±10 %), utilities (95 % confidence intervals assuming beta distribution), distribution of AO risk groups (±20 %) and probabilities of distant recurrence (95 % confidence interval, assuming beta distribution).

In addition, the results of our analysis from an Austrian perspective were compared to a Canadian cost-effectiveness study conducted by the Toronto Health Economics and Technology Assessment (THETA) Collaborative. (Paulden et al. 2013). The THETA group developed and applied a probabilistic Markov model (state-transition cohort model).

For our analysis, 100,000 patients were simulated per strategy, which was tested to provide stable results (Jahn et al. 2015).

## Results

### Base-case analysis

The results of the base-case analysis for Austria are displayed in Table 2. When compared to AO alone (NNN), using ODX in intermediate and high risk patients (NYY) increased discounted life years and QALYs by 2.8 % (NNN: 14.33 LY, 11.08 QALY; NYY: 14.73 LY, 11.40 QALY). When using ODX in all patients (YYY) life years and QALYs increased by 4.1 % (YYY: 14.92 LY, 11.54 QALY). However, costs also increased by 3.9 % (NNN: 13,180 EUR, NYY: 13,710 EUR) to 13.9 % (YYY: 15,930 EUR).

**Table 2:** Cost-effectiveness of ODX in the Austrian setting

Strategy	Base-case analysis				∆Total costs × 1000€	ICER (€/QALY)
	LYs	QALYs	∆QALYs	Total costs ×1000€		
1-NNN	14.33	11.08	–	13.18	–	NA^a^
3-NYN	14.46	11.18	–	13.36	–	D^b^
2-NNY	14.60	11.29	–	13.58	–	D
4-NYY	14.73	11.40	0.32	13.71	0.53	1628.35
5-YNN	14.46	11.17	–	15.48	–	D
7-YYN	14.64	11.31	–	15.61	–	D
6-YNY	14.76	11.41	–	15.81	–	D
8-YYY	14.92	11.54	0.14	15.93	2.22	15,727.78

^a^NA not applicable since NNN is the first comparator

^b^D dominated strategies discounted

The base-case analysis shows that YYY (ODX provided to all patients) is the most effective strategy and is cost effective with an ICER of 15,700 EUR per QALY gained when compared to other technologies in health and medicine. Strategies NNN and NYY are also on the cost-effectiveness frontier but they are less effective. Five of eight strategies were dominated (i.e., more costly and less effective: NNY, NYN, YNN, YNY, YYN).

In Table 3, further information on the benefits and harms of the evaluated strategies are displayed. Here we see that, for example, the strategy NYY leads to the fewest people (0.0301 %) suffering from adverse drug events (ADE). Providing ODX to all patients leads to a higher number of ADE (0.0396 %) compared to NYY but fewer patients develop recurrence (NYY: 0.2051 %; YYY 0.1722 %).

**Table 3:** Additional model outcomes

Strategy	Adverse drug event (%)	Number patients with recurrence (%)	Number of death from recurrence^a^
1-NNN	0.0325	0.2677	26,078
2-NNY	0.0318	0.22828	22,186
3-NYN	0.0308	0.24287	23,636
4-NYY	0.0301	0.20509	19,863
5-YNN	0.0422	0.23507	22,905
6-YNY	0.0417	0.19817	19,290
7-YYN	0.0412	0.21193	20,593
8-YYY	0.0396	0.17215	16,705

^a^Per 100,000 patients

### Sensitivity analysis

**Fig. 2:**
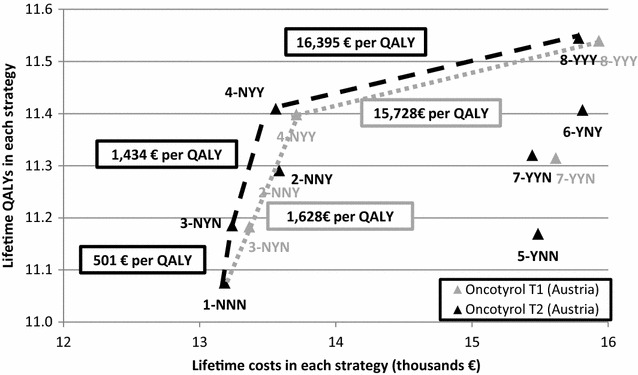
Cost-effectiveness frontier for Austrian test-treatment strategy T1 (base case) and T2 (sensitivity analysis). Sensitivity analysis: risk group AO intermediate/ODX low receive no chemotherapy whereas in the base case 13.73 % would receive chemotherapy, treatment for other risk groups as in base case); ICER – Incremental Cost-Effectiveness Ratios for the not dominated strategies that are on the frontier (ICER = additional costs/additional QALYs for the next most expensive strategy)

In the sensitivity analysis, different adjuvant chemotherapy treatment patterns were considered (Fig. 2). It was assumed that chemotherapy was provided to 13.73 % of patients who had an intermediate risk according to AO and a low risk according to ODX in the base case (T1). In the sensitivity analysis that assumed none of AO intermediate/ODX low patients received chemotherapy (T2), the strategies NYY and YYY remained cost-effective with an ICER comparable to the base case. In addition, the strategy NYN (ICER: 501 EUR/QALY) was cost-effective (Additional file 1: Table S1).

**Fig. 3:**
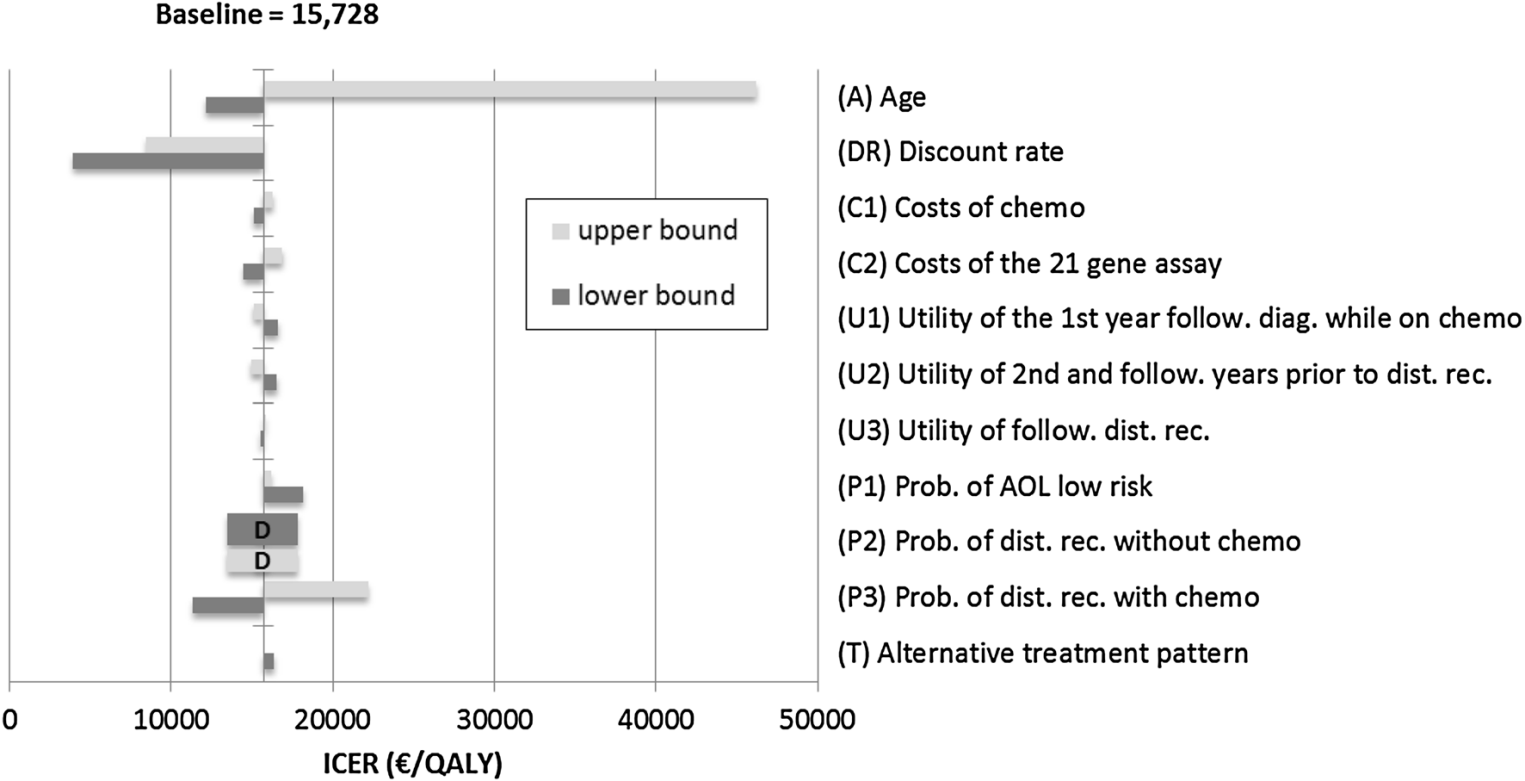
Tornado diagram for the scenario YYY. prob. probability, dist. rec. distant recurrence, follow. following, diag. diagnosis; Parameter range: (A) (40; 50; 70) (DR) (0; 2.5 %; 5 %), (C1) and (C2) ± 10 %, (U1) - (U3) 95 % confidence intervals assuming beta distribution, (P1) ± 20 %, (P2) and (P3) 95 % confidence interval assuming beta distribution,* D* dominated, *AOL* Adjuvant! Online

Further sensitivity analyses found that strategies NYY and YYY remain on the cost-effectiveness frontier with thresholds below 5,300 EUR/QALY (NYY) and 47,000 EUR/QALY (YYY) (Tornado diagram Fig. 3; Additional file 2: Figure S1). Strategy YYY was dominated only when the probability of distant recurrence was increased (upper end of the 95 % confidence interval). The strategy NYN was cost effective when we tested the lower and upper bound of the confidence interval for distant recurrence, 10 % variation in costs, 20 % variation in the AO risk group distribution, a lower discount rate (2.5 %) and younger patient population (40 years). The strategy NNY was cost effective when we tested the upper bound of the confidence interval for distant recurrence for patients who did not receive chemotherapy. All other test-treatment strategies remained dominated (Additional file 1: Table S1).

**Fig. 4:**
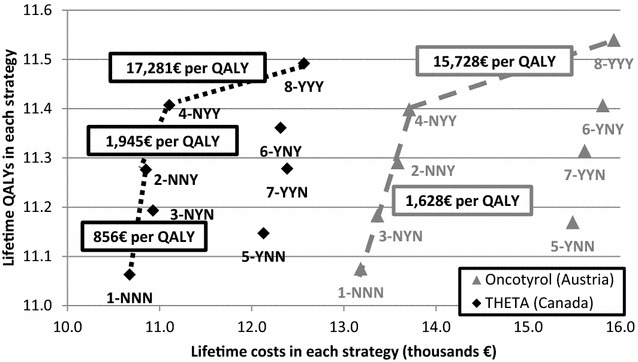
Cost-effectiveness frontier comparison between Austrian and Canadian settings (incl. ICER values)

The comparison of the Austrian base-case analysis and the Canadian analysis by THETA is displayed in Fig. 4.

## Discussion

We developed a decision-analytic model for the evaluation of the 21-gene assay in women diagnosed with ER and/or PR positive, HER-2/neu negative, and lymph node negative breast cancer in Austria. The model is flexible and can be adapted and applied to other countries and health care contexts.

We have demonstrated that the 21-gene assay is a cost-effective tool for determining which ER/PR+, Her2/neu negative early breast cancer patients receive adjuvant chemotherapy when used in two scenarios in the Austrian context; first when used only in patients with a high or intermediate AO risk score (strategy NYY) (ICER 1600 EUR/QALY) and second, when used in all patients independent of the AO risk score (Strategy YYY) (ICER 15,700 EUR/QALY). All other strategies that include testing of low risk AO (YNN, YNY, YYN) are dominated and should not be considered as cost-effective options. With respect to the sensitivity analysis, it seems that the strategy where only AO intermediate risk patients are tested (NYN) could also be a relevant option.

The modeling study shows that the 21-gene assay is cost effective for all AO risk groups. However, there is always the trade-off that the 21-gene assay uses resources that could be used elsewhere. Providing the genetic test to patients with AO intermediate or high risk only would be a cost-effective alternative that uses fewer total resources. However, drivers for the use of the 21-gene assay for AO low risk patients may include disagreements between physicians and patients, uncertainty and anxiety in patients as well as individual health considerations. The time for the genetic testing seems acceptable. The recurrence score is evaluated from a tissue sample sent to Genomic Health within 10–14 calendar days.

The 21-gene assay estimate can differ from the Adjuvant! recurrence estimate for several reasons. The 21-gene assay recurrence estimate is for distant recurrence only (risk of metastatic disease) while AO estimates the risk for all causes of recurrence (local, regional, contralateral breast cancer and distant recurrence). Because of this difference in the endpoint definition, Adjuvant!’s estimates of “risk of recurrence” are usually higher than those of the ODX test. However, the most appropriate comparisons are between the risk of breast cancer mortality as estimated by AO and the risk of distant recurrence as given by the ODX test, though an exact comparison is not possible (Sinn et al. 2013).

Several studies examined the cost effectiveness of ODX driven treatment (Klang et al. 2010; Kondo et al. 2008, 2011; Lyman et al. 2007; Paulden et al. 2013; Reed et al. 2009; Tsoi et al. 2010; Ward et al. 2013; Yang et al. 2012; Hornberger et al. 2005). In the majority of these studies, ODX was found to be superior to conventional risk classification methods (Klang et al. 2010; Kondo et al. 2008, 2011; Lyman et al. 2007; Tsoi et al. 2010; Hornberger et al. 2005). Hall et al. (2012) stated that ODX could be very cost effective for the NHS in the UK, but there is uncertainty in the evidence to support this claim. The Health Technology Assessment by Ward et al. (2013) reported that “compared with current clinical practice, *Oncotype*DX had a 12.4 % (all women) and 91.6 % (Nottingham Prognostic Index >3.4) probability of being considered cost effective when using a threshold of £20,000 per QALY gained respectively”. Yang et al. (2012) compared two different gene expression profiles and concluded that Mammaprint to be even more effective and less costly than ODX. However, these analyses did not consider the provision of ODX conditional upon AO risk and therefore implicitly assumed that AO or conventional risk classification will be replaced rather than combined. Reed et al. (2009) cross-classified patients by clinicopathologic characteristics from AO and ODX. However, they assumed that in the absence of ODX test results, all patients with intermediate and high risk according to AO receive chemotherapy. They did not report test-treatment scenarios where only specific risk groups receive ODX. They estimated an ICER of USD10,788/QALY for an ODX guided therapy as compared to non ODX guided from a US societal perspective. With respect to the Canadian results of the THETA Collaborative, our findings are similar. They identified strategy NYY and YYY to be cost-effectiveness frontier with ICERs a little higher than those for Austria. In addition, in the Canadian setting, strategy NNY is cost effective. The cost-effectiveness frontier showed a similar shape with a slight shift right/up for Austria due to a little higher cost and higher QALYs. This can in part be the result of similar sources of utility data and probabilities of distant recurrence. However, countries differ with respect to chemotherapies that are provided. In addition, for Austria we did not consider end of life care and assumptions of chemotherapy toxicities differ. Hence, it was also found that the strategy NNY is cost effective in the analysis of the THETA collaborative but not for Austria.

Our model has several limitations. Though model parameters were carefully selected, some information was not available for Austria. Therefore, we adapted the distribution of the AO risk groups, utility parameters and estimates for the risk of distant recurrence from international studies (Lo et al. 2010; Paulden et al. 2013; Lidgren et al. 2007). Furthermore, since the 21-gene assay is not available in Austria, the treatment pattern was assumed based on the results of both risk scores as adapted from (Lo et al. 2010) with Austrian experts (Medical University Innsbruck 2012). In general, a review by Carlson and Roth (2013) concluded that there are a lack of studies reporting the impact of ODX on adjuvant chemotherapy use versus a standard approach. Individual health considerations and preferences may be more important in decisions about use of chemotherapy than the results of the 21-gene assay.

There is evidence that ODX results are associated with locoregional recurrence (Mamounas et al. 2010). Within our model, we did not consider the risk of local recurrence. Due to a lack of more detailed data, treatment of distant recurrence was assumed to be similar for all patients independent of the patient history. However, our microsimulation provides the flexibility to incorporate further personalized treatment decisions once that data become available.

ODX, as molecular signatures for ER-positive breast cancer show convincing analytical validity and clinical validity results. However, results have not yet proven robust clinical utility. In some cases, where this decision is difficult, ODX may be used in conjunction with all clinicopathological factors to inform treatment decisions (Azim et al. 2013; Senkus et al. 2013). However, future results from large phase III prospective clinical trials (TAILORx, RxPONDER) may confirm the accurate use of these new tools. Once further data are available, the model will be updated accordingly.

Further tests such as EndoPredict, Breast Cancer Index, MammaPrint, and Genomic Grade Index have shown promising results in ongoing trials. These tests differ slightly with respect to indication, whether they are prognostic or predictive, whether they must be conducted in a central lab, reference lab, or by local pathologists, technique (e.g., qRT-PCR, DNA micro array) outcomes and risk categories. Most tests require formalin-fixed, paraffin embedded tissue (Sinn et al. 2013). The EGAPP working group (EWG) launched by the CDC Office of Public Health Genomics in 2009 (EGAPP Working Group 2009) concluded that the evidence for the use of a gene expression profile is insufficient to assess the balance of benefits and harms for its proposed uses. The EWG encourages further evaluation of these technologies. Likewise, the IMPAKT 2012 Working Group suggested “a need to develop models that integrate clinicopathological factors along with clinical test” (Azim et al. 2013). In addition, Prat et al. (2012) created a dataset of 1380 patients to research the combination of PAM50, ODX, Mammaprint and SET. They showed that combinations of these signatures significantly increase prediction performance. Hence, for future cost-effectiveness studies, comparators and combinations must be thoroughly extended and selected.

Our model can support clinicians in their decision making as to whether the relatively small absolute benefit of chemotherapy in node-negative, ER-positive women outweighs the harm of adverse drug effects. Our discrete event simulation model produced useful results, and is available to be adapted for rapid assessment of new tests, variations in treatment strategies and additional biomarkers and treatments as they become available.

## Conclusion

Our study suggests that ODX applied in all risk groups is the most effective strategy. Also the 21-gene assay is cost effective for patients independent of the AO risk score (Strategy YYY) (ICER 15,700 EUR/QALY) and in patients with a high or intermediate AO risk score (strategy NYY) (ICER 1600 EUR/QALY). Further research is needed to validate the 21-gene assay and use of AO in large prospective studies as well as real world registry studies. The results of future studies of other genetic tests may require alternative or additional test-treatment strategies.

## Additional files

10.1186/s40064-015-1440-6 Results one-way sensitivity analysis
10.1186/s40064-015-1440-6 Tornado diagram for the scenario NYY
