# Supplementary material for: Cost effectiveness of personalized treatment in women with early breast cancer: the application of OncotypeDX and Adjuvant! Online to guide adjuvant chemotherapy in Austria
Source: Springerplus. 2015 Dec 1;4:752. doi: 10.1186/s40064-015-1440-6 (PMC4666888; doi:10.1186/s40064-015-1440-6)
Supplement: Supplementary file 1 — 10.1186/s40064-015-1440-6 Results one-way sensitivity analysis [file 40064_2015_1440_MOESM1_ESM.docx]

**Table S1: Results one-way sensitivity analysis**

| **Strategy** | NYN | | NNY | | NYY | | YYY | |
| --- | --- | --- | --- | --- | --- | --- | --- | --- |
| **Base Case ICER** | **D** | | **D** | | **1,628** | | **15,728** | |
| **Parameter (Range)** | Lower | Upper | Lower | Upper | Lower | Upper | Lower | Upper |
| **Age (**40**; 50;** 70 years**)** | 1,118 | D | D | D | 1,254 | 5,287 | 12,174 | 46,231 |
| **Discount Rate** (0; 2.5; **5**% annually) | D | 369 | D | D | NA | 546 | 3,984 | 8,517 |
| **Costs*:** |  |  |  |  |  |  |  |  |
| Chemo costs (10,236; **11,373;** 12,510 €) | 1,815 | 1,543 | D | D | 1,626 | 1,580 | 15,127 | 16,329 |
| ODX costs (2,862; **3,180;** 3,498 €) | 1,128 | D | D | D | 1,189 | 2,088 | 14,528 | 16,927 |
| **Utilities***:** |  |  |  |  |  |  |  |  |
| 1st year following diagnosis while on chemo (0.509; **0.62**; 0.697) | D | D | D | D | 1,619 | 1,635 | 16,663 | 15,138 |
| 2nd and follow. years prior to dist. recurrence (0.745; **0.779**; 0.811) | D | D | D | D | 1,711 | 1,558 | 16,593 | 14,992 |
| Following dist. recurrence (0.62; **0.685**; 0.735) | D | D | D | D | 1,611 | 1,642 | 15,538 | 15,877 |
| **Probabilities:** |  |  |  |  |  |  |  |  |
| AO low **(0.4239; **0.5299**; 0.6359) | 546 | 181 | D | D | 1,651 | 2,108 | 18,195 | 16,215 |
| distant recurrence no chemo*** | 1,230 | D | D | 2,550 | 1,387 | 3,275 | D | D |
| distant recurrence chemo*** | 985 | 2,691 | D | D | 1,095 | 2,724 | 11,394 | 22,220 |
| **Alternative treatment pattern (T2)** | 501 | | D | | 1,434 | | 16,395 | |

T2: risk group AO intermediate /ODX low receive no chemotherapy whereas in the base case 13.73% would receive chemotherapy, treatment for other risk groups as in base case

Abbreviations: *+/- 10%; **+/- 20%; *** 95% confidence intervals assuming beta distribution

D - dominated strategies. These are strategies that provide fewer QALYs at a higher cost or if there is a more expensive strategy with a lower ICER.

**Sensitivity Analyses**

Table S1 displays the summarized results of all one-way sensitivity analyses. Bold parameter numbers in the first column represent the base case. Lower and upper values are displayed within the parentheses. In columns two to five, we display the results of only those strategies that are not dominated in the base case (NYY, YYY) or in one of the sensitivity analyses (NYN, NNY). The base case results are repeated in the second line; in the following lines, the results for the lower and the upper parameter values are displayed. In summary we see that the results are robust to uncertainties in the utilities. Changes within the other parameters can lead to additional non-dominated strategies and the cost-effective strategies from the base case remain cost-effective with slight changes in the ICERS.
