# Supplementary material for: Cost effectiveness of personalized treatment in women with early breast cancer: the application of OncotypeDX and Adjuvant! Online to guide adjuvant chemotherapy in Austria
Source: Springerplus. 2015 Dec 1;4:752. doi: 10.1186/s40064-015-1440-6 (PMC4666888; doi:10.1186/s40064-015-1440-6)
Supplement: Supplementary file 2 — 10.1186/s40064-015-1440-6 Tornado diagram for the scenario NYY [file 40064_2015_1440_MOESM2_ESM.docx]

Figure S1: Tornado diagram for the scenario NYY


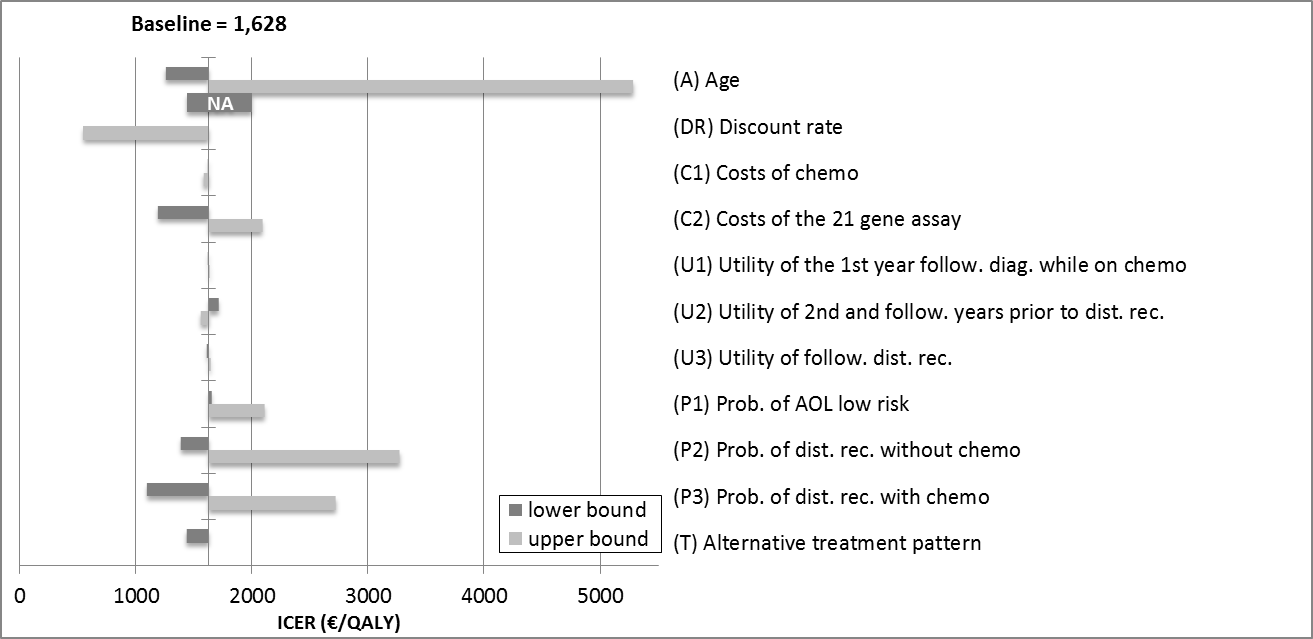


prob. probability, dist. rec. distant recurrence, follow. following, diag. diagnosis, NA not applicable because this strategy is the reference case in the evaluated scenario; Parameter range: (A) (40; 50; 70), (DR) (0; 2.5%; 5%), (C1) and (C2) +/- 10%, (U1) - (U3) 95% confidence intervals assuming beta distribution, (P1) +/- 20% , (P2) and (P3) 95% confidence interval assuming beta distribution)

Figure S1 displays the tornado diagram of the sensitivity analyses for the test-treatment strategy NYY
